# PD-L1 Multiplex and Quantitative Image Analysis for Molecular Diagnostics

**DOI:** 10.3390/cancers13010029

**Published:** 2020-12-23

**Authors:** Fatima Abdullahi Sidi, Victoria Bingham, Stephanie G. Craig, Stephen McQuaid, Jacqueline James, Matthew P. Humphries, Manuel Salto-Tellez

**Affiliations:** 1 Precision Medicine Centre of Excellence, The Patrick G Johnston Centre for Cancer Research, Queen’s University, Belfast BT9 7AE, UK; F.AbdullahiSidi@qub.ac.uk (F.A.S.); v.bingham@qub.ac.uk (V.B.); Stephanie.Craig@qub.ac.uk (S.G.C.); s.mcquaid@qub.ac.uk (S.M.); j.james@qub.ac.uk (J.J.); m.humphries@qub.ac.uk (M.P.H.); 2 Cellular Pathology, Belfast Health and Social Care Trust, Belfast City Hospital, Lisburn Road, Belfast BT9 7AB, UK; 3 Northern Ireland Biobank, The Patrick G Johnston Centre for Cancer Research, Queen’s University, Belfast BT9 7AE, UK

**Keywords:** PD-L1, high-throughput workflow, multiplexing, image analysis

## Abstract

**Simple Summary:**

Assessing some diagnostic tests can be extremely difficult, even for highly trained clinicians. We have shown in the past that by using an advanced computer software program (QuPath), applied to high resolution images of patient tissue samples, we can assist pathologists in their assessment of a routine test that determines immunotherapy treatment. We also showed that by using a different testing method in the laboratory, called multiplexing, which detects several proteins at once rather than just one alone, we are subjectively more confident in the patient’s reported score. Here, we show that multiplexing is comparable to the traditional method, and that we can also easily apply our computer software tools to extract very specific information from the patient samples, which we are unable to do using the traditional laboratory method. We believe these tools can support pathologists to triage patient cases for this important diagnostic test.

**Abstract:**

Multiplex immunofluorescence (mIF) and digital image analysis (DIA) have transformed the ability to analyse multiple biomarkers. We aimed to validate a clinical workflow for quantifying PD-L1 in non-small cell lung cancer (NSCLC). NSCLC samples were stained with a validated mIF panel. Immunohistochemistry (IHC) was conducted and mIF slides were scanned on an Akoya Vectra Polaris. Scans underwent DIA using QuPath. Single channel immunofluorescence was concordant with single-plex IHC. DIA facilitated quantification of cell types expressing single or multiple phenotypic markers. Considerations for analysis included classifier accuracy, macrophage infiltration, spurious staining, threshold sensitivity by DIA, sensitivity of cell identification in the mIF. Alternative sequential detection of biomarkers by DIA potentially impacted final score. Strong concordance was observed between 3,3’-Diaminobenzidine (DAB) IHC slides and mIF slides (R^2^ = 0.7323). Comparatively, DIA on DAB IHC was seen to overestimate the PD-L1 score more frequently than on mIF slides. Overall, concordance between DIA on DAB IHC slides and mIF slides was 95%. DIA of mIF slides is rapid, highly comparable to DIA on DAB IHC slides, and enables comprehensive extraction of phenotypic data and specific microenvironmental detail intrinsic to the sample. Exploration of the clinical relevance of mIF in the context of immunotherapy treated cases is warranted.

## 1. Introduction

Extensive exploration into the interaction between programmed cell death protein-1, (PD-1) and programmed death-ligand 1 (PD-L1) within the tumour microenvironment has led to significant breakthroughs in the treatment of malignancies associated with these immune checkpoint proteins [1,2]. However, challenges still exist in the determination of appropriate techniques used in the molecular diagnostic quantitation of these biomarkers, especially in non-small cell lung cancer (NSCLC). There are several FDA-approved tests that are routinely used clinically, which comprise different antibody clones as the principal tools for detecting the PD-L1 biomarker, for example the Dako PD-L1 22C3 clone is specifically used for directing treatment with the anti-PD-1 agent pembrolizumab or the Ventana PD-L1 SP142 clone for the prescription of atezolizumab, both in NSCLC as well as other tumour types. There are several challenges faced with the assessment of these techniques, as highlighted in the literature [3,4,5,6].

The mainstay of routine clinical diagnostics is centred on the microscopic review of haematoxylin and eosin (H&E) and chromogenic immunohistochemistry (IHC) slides. Nevertheless, the advent of whole slide image scanning and the on-screen digital representation of tissue slides has facilitated the application of digital image analysis (DIA) to augment pathological assessment. In addition, increasing access to robust automated multiplex immunofluorescence (mIF) assays and whole-slide fluorescent imaging increasingly represents an attractive direction of travel for tissue-based molecular diagnostics, due to the extraordinary ability to accurately interrogate the tumour microenvironment. The capability to detect multiple biomarkers in distinct topographical tissue compartments may represent the dawning of a new revolution in surgical pathology, especially concerning molecular diagnostics and particularly in the era of cancer immunotherapy.

In an effort to improve the diagnostic workflow and augment the pathologist, the use of DIA has been applied to chromogenic digital images [4,5,7,8]. We recently reported highly concordant data comparing manual assessment of PD-L1 and DIA assessment, which yielded a significant degree of accuracy on IHC slides [4,9]. Further to this, it has been reported that mIF can improve the diagnostic accuracy in determining PD-L1 positivity within tumour cells to a higher degree than IHC [4,5,10,11].

Studies are emerging that increasingly show that immune profiling and biomarker co-localisation may greatly improve immunotherapeutic strategies and these can be achieved largely by employing mIF methods [11,12,13]. Advancements in multiplexing technology bring the potential to deploy mIF in a clinical setting [4,14,15]. Studies are also beginning to show that applying DIA to mIF can significantly improve biomarker detection [11,16,17].

The adoption of mIF, particularly if aligned with robust DIA, could represent a significant advancement in the workflows of clinical diagnostic departments and augment the pathologist in the decision-making process of challenging cases. Harmonising the technical complexities of mIF in the laboratory with the speed of DIA is essential for translation into routine diagnostic setting to help potentially triage case workflow, as we have proposed [4].

In the present study, we used an optimised automated mIF staining protocol developed for the assessment of the immune checkpoint marker PD-L1, an epithelial cell marker cytokeratin (CK), a macrophage marker (CD68), a T-cell marker (CD8), and a nuclear stain (DAPI), coupled with a high throughput image analysis pipeline. Our goal was to rapidly and specifically assess PD-L1 positivity in PD-L1+/CK+ cells or PD-L1-/CK+ cells, while discounting potential confounding PD-L1+/CD68+ macrophages and PD-L1+/CD8+ immune cells.

## 2. Results

### 2.1. Biomarker Optimisation and Validation

**Figure 1:**
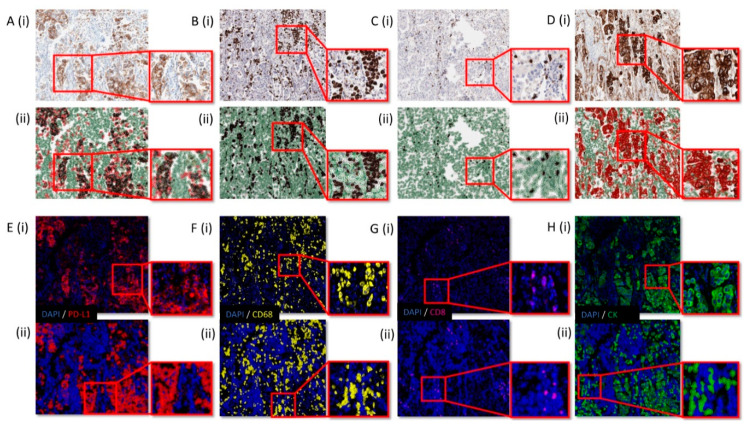
Optimised chromogenic immunohistochemistry (IHC) and single channel multiplex immunofluorescence (mIF) staining. (**A**–**D**) (**i**) represents optimised DAB IHC for PD-L1, CD68, CD8, and CK, respectively. (**A**–**D**) (**ii**) represents the QuPath image analysis mask applied to the optimised DAB IHC images. In (**A**) (**ii**), the tumour class is coloured red, while stroma is green. PD-L1 positive cells are black within the red tumour class. In (**B**) (**ii**), positive CD68 cells are black and negative CD68 cells are green. In (**C**) (**ii**), positive CD8 cells are black and negative CD8 cells are green. In (**D**) (**ii**), positive CK cells are red and negative CK cells are green. (**E**–**H**) (**i**) represent optimised single fluorescence Opal staining for PD-L1, CD68, CD8, and CK, respectively. (**E**–**H**) (**ii**) represents the QuPath image analysis mask applied to the optimised DAB IHC images. In (**E**) (**ii**), PD-L1 positive cells are red with all other cells blue. In (**F**) (**ii**), positive CD68 cells are yellow and negative CD68 cells are blue. In (**G**) (**ii**), positive CD8 cells are purple and negative CD8 cells are blue. In (**H**) (**ii**), positive CK cells are green and negative CK cells are blue. All images are 10× magnification with an ROI shown as an exploded view at 20× magnification.

Following optimisation of singleplex chromogenic IHC staining, using established methods we have reported previously [18], (Figure 1: A (i), PD-L1; B (i), CD68; C (i), CD8; and D (i), CK), manual assessment of single Opal fluorescence channels in the multiplex were conducted to ensure accurate reflection of the 3,3’-Diaminobenzidine (DAB) IHC (Figure 1: E (i), PD-L1; F (i), CD68; G (i), CD8; and H (i), CK). The raw image and the digital pathology cell detection masks are shown in Figure 1(i),(ii), respectively. The order of staining was then optimised to ensure accurate recapitulation of the DAB following multiple epitope retrieval rounds, each step was visually assessed and was determined to be highly representative by experienced individuals with an expertise in the assessment of chromogenic and fluorescent staining.

**Figure 2:**
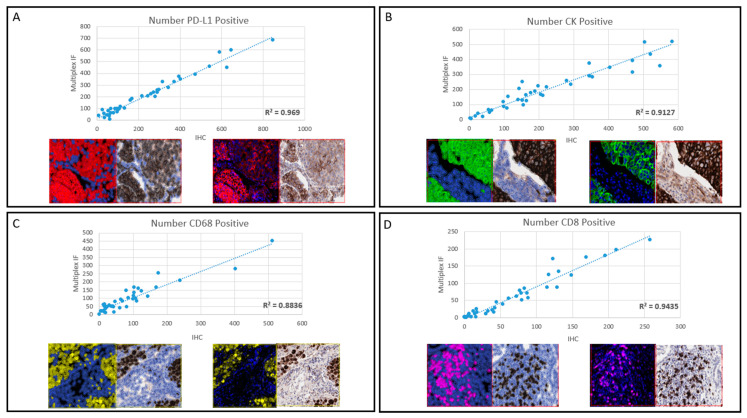
Biomarker concordance between the DAB IHC and the singleplex Opal fluorescence. Each panel displays a graph showing the single marker IF on the *y*-axis and the DAB IHC and the *x*-axis, for the number of cells detected. The image analysis mask overlaid on the mIF and IHC images are shown on the bottom left, with the native mIF and IHC image shown on the bottom right. Panel (**A**): PD-L1. Panel (**B**): CK. Panel (**C**) CD68. Panel (**D**): CD8. R^2^ are displayed in the graphs. Images are ×20 magnification.

A DIA approach was then undertaken to specifically quantify the comparability of the like-for-like staining between the traditional DAB IHC and the optimised fluorescent single-channel from the multiplex panel, for each biomarker in sequence. The optimised sequence is shown in Supplementary Table S1. For each biomarker, on both the DAB IHC and single channel mIF digital scans, 40 matched regions of interest (ROIs) were digitally annotated in nine cases demonstrating expression of all relevant biomarkers, totalling 160 ROIs. Each patient, dependant on the availability of the tissue, had 17–18 ROIs annotated on the digital image, of the 17–18, 4–5 ROIs for each biomarker were captured. Digital quantification revealed that the like-for-like expression of each biomarker within each ROI was not significantly different between the DAB IHC and the singleplex Opal fluorescence (Figure 2). Across all ROIs, the expression of each biomarker was strongly correlated for both techniques. Data for total cell number are reported with representative images for each biomarker. The highest correlation between DAB IHC and mIF was seen for PD-L1, while the lowest correlation was CD68. For PD-L1, the correlation was R^2^ = 0.969, CK correlation R^2^ = 0.912, the correlation for CD68 was R^2^ = 0.883, and CD8 correlation was R^2^ = 0.943.

### 2.2. Multiplex Combination and PD-L1 Phenotypic Assessment

**Figure 3:**
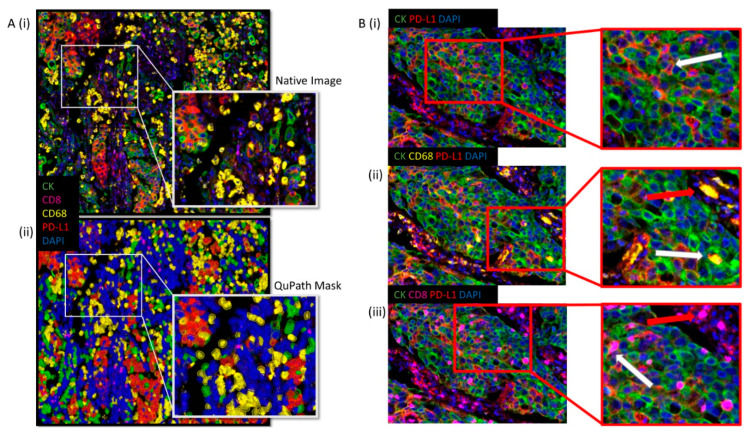
Optimised digital image analysis (DIA) assessment of the mIF staining. (**A**) (**i**) represents the composite mIF staining panel with nuclei (blue), PD-L1 (red), CK (green), CD68 (yellow), CD8 (purple). (**A**) (**ii**) displays the QuPath mask of the combined multiplex with nuclei (blue), PD-L1 (red), CK (green), CD68 (yellow), CD8 (purple). (**B**) (**i**) displays only PD-L1 positive, cytokeratin positive channels, while (**B**) (**ii**) and (**iii**) display the addition of CD68 and CD8, respectively.

The combined multiplex for PD-L1, CK, CD68, CD8, and DAPI was applied to the tissue microarray (TMAs). mIF staining was sensitive, specific, and each channel was found to be representative of the staining pattern seen by DAB IHC, Figure 3(Ai).

Detection of biomarkers in a specific sequence was essential to enable accurate extraction of relevant phenotypic PD-L1 expression data. The optimised method for cell identification was as follows: All cells were detected using the DAPI channel, detecting every nucleated cell. A tumour classifier was created using a threshold for positivity using the CK channel only. Phenotyping of CD8-positive cells, followed by CD68-positive cells, was conducted, which facilitated the removal of potential confounding cells that may be PD-L1 positive and located within the tumour epithelial nests. Within the tumour class (CK positive), PD-L1 positive or negative cells were then identified within the CK positive tumour cells only. The optimised DIA assessment and QuPath mask of the combined multiplex is shown in Figure 3(Aii) and delineates the range of phenotypic expression of each cell type as described. A flow diagram of the optimised method for cell identification is shown in Supplementary Figure S1.

The power of multiplexing to make available only precise biomarker observations is shown in Figure 3B, where, B (i) displays only PD-L1-positive, cytokeratin-positive channels, providing the observer the ability to identify PD-L1 positive expression distinctly within or separate from tumour epithelial nests. With the added observation of CD68, Figure 3B(ii), macrophages can be readily identified as intratumoural or not, as indicated. Similarly, observation of CD8 enables the observer to identify the topographical localisation of T-cells, Figure 3B(iii).

To eliminate the influence of PD-L1-positive CD68 macrophages and/or CD8 T-cells on the overall PD-L1 score, CD68 and CD8 cells were identified initially by digital analysis followed by the classification of tumour. This was necessary due to the proximity of the CD68 and CD8 cells to CK, which were often within the CK positive tumour epithelial nests. The requirement to recognise CD68 cells early in the image analysis process was identified, after several sequence investigations. It was determined that detection of the PD-L1 signal as the initial analysis channel led to an inaccurate, overinflated, final score, as shown in Supplementary Figure S2. This is explicable as a cell detected as PD-L1-positive may also have been either CD68- or CD8-positive or negative and must first be assigned that phenotype before assessing the PD-L1 channel. These detection processes influenced either the numerator or denominator when calculating the percentage tumour PD-L1 positivity. Upon agreement of the optimised detection sequence, which accurately enabled the extraction of PD-L1-positive epithelial cells, while discounting PD-L1 positive macrophages and T-cells, a bespoke script was created and utilised across all mIF slides.

### 2.3. Image Analysis Correlation of mIF and IHC

**Figure 4:**
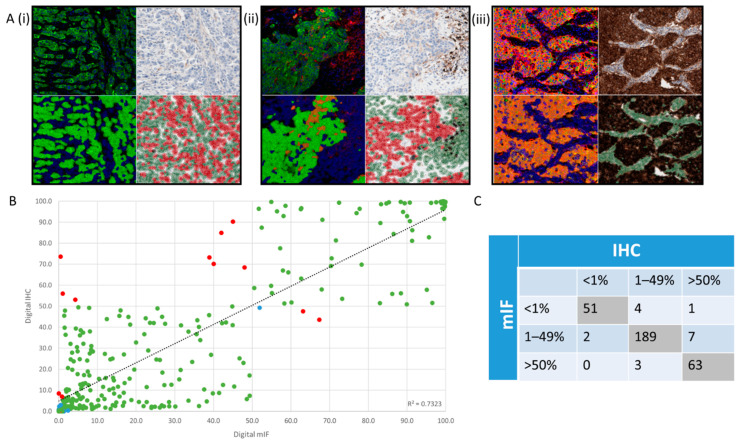
PD-L1 concordance between mIF and DAB IHC. Clinical categories, <1%, 1–49%, or >50%, are shown by representative mIF images (**A**) (**i**–**iii**), respectively. Each panel shows mIF image (top left), including the DIA mask (bottom left) and a DAB image (top right), including the DIA mask (bottom right). (**B**) shows DIA percentage positivity on 320 mIF TMA cores compared to DIA percentage positivity of the corresponding DAB IHC slides (R^2^ = 0.7323). Green data points display concordance, red display discordance, and blue demonstrate cases where the ground truth is difficult to discern upon review. (**C**) displays the raw categorical data displaying the graph in (**B**).

Taking the optimised bespoke mIF detection algorithm, which extracts the phenotypic expression of PD-L1 in CK+ tumour cells only, after removing CD68+/PD-L1+ and CD8+/PD-L1+ cells, all 320 TMA cores were scored for PD-L1 tumour cell expression. All DAB IHC images for 320 TMA cores were also assessed for PD-L1 expression by DIA, as described in [4]. These separate scoring metrics were extracted from QuPath for each image type, DAB and mIF, respectively, and each hybridisation method correlated based on clinical category. Concordance was established if both PD-L1 scores resulted in the same clinical category (<1%, 1–49% or >50%), as shown by representative mIF and DAB images in Figure 4(Ai–iii), respectively. Figure 4B shows that applying DIA on 320 mIF TMA cores yielded highly concordant results when compared to DIA of the corresponding DAB IHC slides (R^2^ = 0.7323), with Figure 4C displaying the raw data.

In 12 instances, we observed that DIA on DAB IHC slides resulted in an overcall of the PD-L1 score with significant difference from the mIF to shift the clinical category, while this observation was seen only five occasions for mIF. These discrepancies constituted 5.3% (17/320) of the study cohort. Yet overall, the concordance between DIA on DAB IHC slides and mIF slides was 94.7% (303/320).

**Figure 5:**
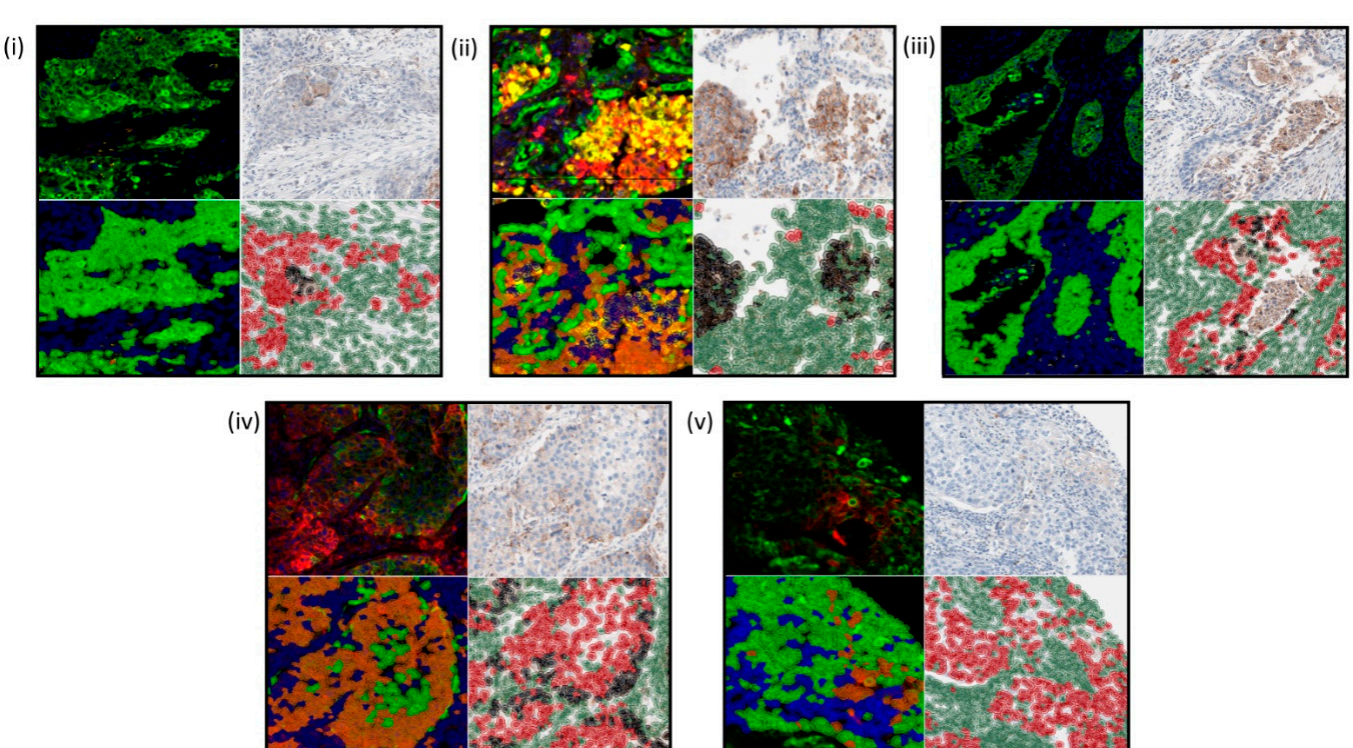
Reasons for discordance. These were due to several factors. (**i**) Classifier inaccuracy on the DAB slide, (**ii**) macrophage infiltration in both image types, (**iii**) spurious staining in both image types, (**iv**) threshold sensitivity in both image types, (**v**) sensitivity of cell identification in the mIF.

The strong correlation between both methods demonstrates that for the majority of assessments, DIA is a sufficient method of automated analysis. In the 5.3% (*n* = 17) of discordant assessments, a thorough review of these comparisons was undertaken. For 70.6% (*n* = 12) of these cases it was determined that DIA conducted on mIF slides provided a higher degree of subjective confidence in the overall score than that of DAB IHC images. Additionally, the reasons for discordance were due to several factors, which should be considered when assessing mIF. (1) Classifier inaccuracy on the DAB slide, (2) macrophage infiltration in both image types, (3) spurious staining in both image types, (4) threshold sensitivity in both image types, (5) sensitivity of cell identification in the mIF. Examples of these factors are shown in Figure 5i–v, respectively.

## 3. Discussion

The ability of DIA to aid diagnostic interpretation of slides is challenging the paradigm in surgical pathology. Evolving away from subjective and time-consuming processes, whereby the intensity of staining, localisation, and quantity are assessed manually. DIA brings a rapid, numerically quantifiable and reproducible workflow. We have previously shown that DIA may improve the PD-L1 diagnostic workflow when applied to chromogenic digital images [9]. We also demonstrated digital assessment of PD-L1 DAB slides was comparable to manual pathological assessment [4]. Furthermore, we described the utility and beneficial application of mIF in challenging molecular diagnostic cases of NSCLC to aid the pathologist in the manual assessment of PD-L1 [4]. Here, we established that DIA applied to mIF slides is a viable methodology for the generation of accurate and reliable quantification of PD-L1 positivity, in comparison to DIA of DAB IHC, in NSCLC. We highlighted the necessity of prevalidation of single biomarkers, prior to combination into multiplex, and importantly, the consideration for a logical sequence of digital assessment.

The high correlation and reproducibility of biomarker data generated between DAB and mIF is fundamental in the establishment of mIF as a robust method of biomarker interrogation. Several studies have reported their experience with this assessment, and while our data, and those reported previously by our group [17], indicate that single mIF staining is highly correlated with IHC, some provide only anecdotal evidence [19]. Koelzer et al. reported comparable staining between mIF chromogenic IHC staining in their panel (PD-L1, CD3, CD8, CD57, and PD-1) [15]. Not all reports provided such compelling comparative data. Parra et al. reported variable cell expression during their staining of mIF across different carcinoma tissues batches, which is an observation we have not encountered. In addition, their observations, while significant, reported R^2^ correlations as low as 0.527. It is worth noting that in these studies and our own, the mIF panels developed utilised Opal reagents based on tyramide signal amplification staining technique, which is known to be more sensitive than conventional fluorescence or DAB IHC [20] and could account for the less than perfect comparisons. Though it is conceivable that robust biomarkers that are not scored based on gradient contribute to the higher accuracy studies.

The sensitivity of the tyramide signal amplification staining technique could explain some of the observations we have seen when assessing PD-L1 by mIF, where we have seen an increased sensitivity in the mIF method to detect PD-L1. This has occasionally resulted in disparate overall scores in comparison to the DAB that gives rise to a change in the clinical category determined for the sample. However, in our hands, we observed an excellent correlation when DAB IHC and mIF were quantified digitally in ROIs assessing like-for-like tissue staining. It remains to be determined whether this increased sensitivity has any impact on clinical response to anti-PD-1 inhibitors, but recent data indicate that PD-L1 assessment by mIF yields improved clinical performance to immunotherapy [10].

When extracting PD-L1 expression data from regions of tumour epithelium, there are considerations and potential confounding factors highlighted in our study that are shared across both mIF and DAB IHC methodologies. We report that macrophage infiltration and the sensitivity of the detection threshold can confound the DIA for mIF and DAB, as we have previously commented on for DIA in DAB images [4]. The classification inaccuracy seen on DAB IHC images was not observed in mIF images, this can be reasoned due to the epithelium being specifically identified by CK, allowing for a much more precise tumour classifier creation. Moreover, though our clinical categorical correlations are compelling, it is interesting to note a degree of overcall of the PD-L1 score by DIA on the DAB images. An observation we observed in our previous study [4], where DIA was found to overcall the PD-L1 score relative to the pathologists’ manual assessment. It is tempting to speculate that the lack of overcall by mIF is owed to its ability to delineate multiple cells types within the specimen and thus more closely match the real-world method of assessment as a pathologist. However, we also found mIF occasionally suffered an increase sensitivity of cell identification in the sample, the implication for DIA was therefore a detection of an increased number detected in the DAPI channel. This too may also lend more confirmation to the evidence that tissue thickness should be considered in any mIF study design, as we have noted in a previous report when considering the impact of autofluorescence [17]. While the comparative PD-L1 score was highly correlated between DAB and mIF, the increased sensitivity of the mIF technique may account for the lower number of overcalls in comparison to DIA in DAB IHC. That being said, there is little doubt of the consistency and accuracy of DIA, which is borne-out in the improvements in the prognostication and predictive stratification of data generated [21].

In our experience with the clinical assessment of PD-L1, our observations regarding the correlations of manual pathologist scoring with DIA on DAB IHC slides [4], coupled with the DAB IHC and mIF concordance reported here, we strongly advocate the use of DIA in biomarker quantitation, as hypothesised in our earlier work [9]. We are confident in the utility of DIA in both a research and clinical setting to draw out the phenotypic data required in the vast majority of specimens to accurately ascertain PD-L1 epithelial cell expression.

## 4. Materials and Methods

### 4.1. Patient Samples

Access to a series of sequential 3 µm NSCLC formalin-fixed paraffin-embedded (FFPE) TMA sections was granted under the Northern Ireland Biobank (NIB) approval (NIB15-0168). NIB has ethical approval to use deidentified tissue samples from the Belfast Health and Social Care Tissue Pathology archive (REC:11/NI/0013) [22]. These TMA sections contained 320 1 mm cores available for analysis, from 56 cases, which were an even mix of adenocarcinomas and squamous cell carcinomas. These cases were a subset of cases from our previous study [4], arranged in TMA format.

### 4.2. Routine Diagnostic Immunohistochemistry

Single-plex chromogenic IHC was performed using an automated staining system (Ventana BenchMark) with PD-L1 SP263 clone dispensed neat via a locked-in protocol, as recommended by the company (Ventana, CC1 pre-treatment for 64 min, Ventana Optiview detection protocol), a diaminobenzidine (DAB) reaction was used to detect antibody labelling with haematoxylin counterstaining. To assess specificity and sensitivity, an intra-run reproducibility section from a four core TMA was used in each test run, representing PD-L1 expression levels of <1%, 1–49%, and >50%, as well as a positive control (tonsil). All clinically assessed cases were scored by teams of two individuals who received training and are certified competent for PD-L1 scoring. All DAB IHC slides were scanned at 40x on an Aperio AT2 digital scanner (Leica Biosystems, Milton Keynes, UK). Images were automatically stored on a secure network server.

### 4.3. Multiplex Immunofluorescence Panel Validation

Validation of the staining sequence was conducted to accurately control the specific conditions applied in the multiplex, as described [4,17]. This included the sequential ordering of primary antibodies to ensure representative epitope stability and therefore comparable expression of singleplex DAB staining vs. singleplex IF staining following several rounds of epitope retrieval. Optimised retrieval methods, staining conditions, and steps were conducted according to the manufacturer’s instructions and detailed in Supplementary Table S1. Following validation, biomarkers were paired with Opal secondaries. Combination of all biomarkers in the multiplex was then conducted and validated by an expert panel.

This specific mIF panel was performed according to established protocols previously described [4,9]. Briefly, using an Opal 7-Color Automation IHC Kit (Akoya Biosciences, Marlborough, MA), an optimised multiplex for PD-L1, CK, CD68, CD8, and DAPI was conducted on a Leica Bond Rx fully automated immunostainer. Biomarkers that could co-localise in the same cellular compartment were paired with a spectrally separated Opal fluorophore to avoid potential spectral interference, as recommended by the manufacturer. PD-L1 SP263 was diluted 1:2 from the Ventana BenchMark ready to use cartridge. Antibody retrieval information and Opal pairings information is available in Supplementary Table S1. All mIF slides were scanned on an Akoya Vectra Polaris (Akoya Biosciences, Marlborough, MA) at ×20 using MOTiF™ protocol, which generates a single unmixed whole slide scan of up to 7 colours. This single image facilities a rapid application of DIA across the entire slide in a streamlined workflow, without the requirement of stitching many spectrally unmixed image tiles. Images were automatically stored on a secure networked server for ready access by image analysts.

### 4.4. Serial Chromogenic and Single Immunofluorescence Staining

On sequential TMA sections, each biomarker was stained by either chromogenic IHC for PD-L1, CK, CD68, or CD8, or the optimised mIF panel for PD-L1, CK, CD68, CD8, and DAPI. Optimised retrieval methods and staining conditions are detailed in Supplementary Table S1, and were as described by [4,17]. CK was applied to serial Section 1; PD-L1 to serial Section 2; mIF panel for PD-L1, CK, CD68, CD8, and DAPI to serial Section 3; CD68 to serial Section 4; and CD8 to serial Section 5. DAB IHC slides were scanned at ×20 on an Aperio AT2 digital scanner (Leica Biosystems, Milton Keynes, UK) and mIF slides were scanned on an Akoya Vectra Polaris (Akoya Biosciences, Marlborough, MA, USA) at ×20 using the MOTiF™ protocol. Images were automatically stored on a secure networked server. Comparable quantitation of single channel mIF with the DAB IHC on sequential sections was undertaken by DIA.

### 4.5. Image Analysis

Aperio .svs DAB or Akoya .qptiff fluorescence images were imported in to QuPath (version 0.2.0-m11) from the secure networked server [23]. Following a method of TMA dearraying, a rigorous quality control process was undertaken by an experienced image analyst to remove necrosis, tissue folds and normal epithelium that may confound the analysis. This was confirmed by a second reviewer with frequent consultations with an experienced pathologist prior to analysis, as previously reported [4,9,13,17,24,25].

For IHC DAB, cell detection was conducted using the haematoxylin channel for H-DAB slide types using default parameters. Classification of cell types was conducted using training annotations (MH/FAS/SC) to train a random forest classifier to distinguish tumour and stroma compartments, under the consultation of pathologists experienced in PD-L1 clinical assessment (SMcQ/JJ/MST). A PD-L1 positive tumour cell was defined as a tumour epithelial cell with membrane DAB staining of any intensity (partial or complete). Within QuPath, this was detected by classification by specific feature, within the class (tumour), above a DAB threshold of 0.15, as previously reported [4,9]. For IHC biomarkers across serial TMA sections for CK, CD68, and CD8, positive cell detection was used to identify positive cells within the annotated tissue.

For mIF, cell detection was conducted using the DAPI channel. Correlations of single channel fluorescence were compared to the single IHC biomarkers in 40 matched ROIs assessing like-for-like staining. Single channel fluorescence detection was carried out using the positive cell detection feature based on agreed thresholds for each biomarker CK, PD-L1, CD68, and CD8. Correlation data was statistically analysed by Spearman’s rank coefficient.

DIA of the combined multiplexed biomarkers assessing the phenotypic expression of PD-L1 was conducted by application of a bespoke script created within QuPath for the detection of the individual biomarkers using their corresponding thresholds, as well generating the PD-L1+/CK+ cell count, expressed as a percentage of overall CK positive tumour cells (Supplementary Data S1). A PD-L1 positive tumour cell was defined in mIF as CK positive with partial or complete PD-L1 membrane staining.

## 5. Conclusions

At a time when the global pathology community is under constant and ever-increasing pressure, we bring to bear multiple digital pathology tools and tissue hybridisation techniques with the goal of augmenting the pathologist to deliver specific, reliable, and high-quality reporting. Taken together, with our studies [4,9] and data presented here, we recommend a stepwise process of sample triage to increase throughput and support the pathological assessment of PD-L1. This can be achieved through a combination of DAB IHC and mIF assessment with a DIA backbone across our digital pathology workflows. We confidently propose based on our expertise with PD-L1 assessment, and the work of others, that DIA be readily applied to all DAB IHC slides, and in cases close to clinical thresholds, multiplexing is recommended followed by the application of rapid and seamless digital quantitation [4,9,10,17].

The barriers to the successful deployment of an end-to-end workflow are not small. Specialised centres of excellence, with access to state of the art equipment and expertise are required to successfully deliver such a workflow. However, it is important to see that tools able to quantitate complex signals are robust enough to be eventually validated for diagnostic purposes and for accreditation-driven clinical trial analysis.

## Data Availability

The data is part of a current research application for which IP protection is required. As such, the data presented in this study may be available on request from the corresponding author, subject to IP protection agreements.

## Supplementary Materials

The following are available online at https://www.mdpi.com/2072-6694/13/1/29/s1, Figure S1: Flow diagram for mIF cell detection, Figure S2: Importance of detection order, Table S1: Optimised retrieval methods and staining conditions, Data S1: Bespoke Script for the Detection of Individual Biomarkers on mIF Images Using Qupath.

## References

[1] Smyth M.J., Ngiow S.F., Ribas A., Teng M.W.L. (2016). Combination cancer immunotherapies tailored to the tumour microenvironment. Nat. Rev. Clin. Oncol..

[2] Wang D.Y., Ye F., Zhao S., Johnson D.B. (2017). Incidence of immune checkpoint inhibitor-related colitis in solid tumor patients: A systematic review and meta-analysis. OncoImmunology.

[3] Widmaier M., Wiestler T., Walker J., Barker C., Scott M.L., Sekhavati F., Budco A., Schneider K., Segerer F.J., Steele K. (2020). Comparison of continuous measures across diagnostic PD-L1 assays in non-small cell lung cancer using automated image analysis. Mod. Pathol..

[4] Humphries M.P., Bingham V., Sidi F.A., Craig S.G., McQuaid S., James J., Salto-Tellez M. (2020). Improving the Diagnostic Accuracy of the PD-L1 Test with Image Analysis and Multiplex Hybridization. Cancers.

[5] Gupta S., Zugazagoitia J., Martinez-Morilla S., Fuhrman K., Rimm D.L. (2020). Digital quantitative assessment of PD-L1 using digital spatial profiling. Lab. Investig..

[6] Rimm D.L., Han G., Taube J.M., Yi E.S., Bridge J.A., Flieder D.B., Homer R., West W.W., Wu H., Roden A.C. (2017). A Prospective, Multi-institutional, Pathologist-Based Assessment of 4 Immunohistochemistry Assays for PD-L1 Expression in Non–Small Cell Lung CancerAssessment of 4 Assays for PD-L1 Expression in NSCLCAssessment of 4 Assays for PD-L1 Expression in NSCLC. JAMA Oncology.

[7] Schaumberg A.J., Rubin M.A., Fuchs T.J. (2018). H&E-stained Whole Slide Image Deep Learning Predicts SPOP Mutation State in Prostate Cancer. bioRxiv.

[8] Martinez-Morilla S., McGuire J., Gaule P., Moore L., Acs B., Cougot D., Gown A.M., Yaziji H., Wang W.-L., Cartun R.W. (2020). Quantitative assessment of PD-L1 as an analyte in immunohistochemistry diagnostic assays using a standardized cell line tissue microarray. Lab. Investig..

[9] Humphries M.P., McQuaid S., Craig S., Bingham V., Maxwell P., Maurya M., McLean F., Sampson J., Higgins P., Green C. (2019). Critical appraisal of PD-L1 reflex diagnostic testing: Current standards and future opportunities. J. Thorac. Oncol..

[10] Lu S., Stein J.E., Rimm D.L., Wang D.W., Bell J.M., Johnson D.B., Sosman J.A., Schalper K.A., Anders R.A., Wang H. (2019). Comparison of Biomarker Modalities for Predicting Response to PD-1/PD-L1 Checkpoint Blockade: A Systematic Review and Meta-analysis. JAMA Oncol..

[11] Yeong J.P.S., Tan T., Chow Z.L., Cheng Q., Lee B., Seet A., Lim J.X., Lim J.C.T., Ong C.C.H., Thike A.A. (2020). Multiplex immunohistochemistry/immunofluorescence (mIHC/IF) for PD-L1 testing in triple-negative breast cancer: A translational assay compared with conventional IHC. J. Clin. Pathol..

[12] Lee C.-W., Ren Y.J., Marella M., Wang M., Hartke J., Couto S.S. (2020). Multiplex immunofluorescence staining and image analysis assay for diffuse large B cell lymphoma. J. Immunol. Methods.

[13] Humphries M.P., Craig S.G., Kacprzyk R., Fisher N.C., Bingham V., McQuaid S., Murray G.I., McManus D., Turkington R.C., James J. (2020). The adaptive immune and immune checkpoint landscape of neoadjuvant treated esophageal adenocarcinoma using digital pathology quantitation. BMC Cancer.

[14] Hofman P., Badoual C., Henderson F., Berland L., Hamila M., Long-Mira E., Lassalle S., Roussel H., Hofman V., Tartour E. (2019). Multiplexed Immunohistochemistry for Molecular and Immune Profiling in Lung Cancer—Just About Ready for Prime-Time?. Cancers.

[15] Parra E.R., Uraoka N., Jiang M., Cook P., Gibbons D., Forget M.-A., Bernatchez C., Haymaker C., Wistuba I.I., Rodriguez-Canales J. (2017). Validation of multiplex immunofluorescence panels using multispectral microscopy for immune-profiling of formalin-fixed and paraffin-embedded human tumor tissues. Sci. Rep..

[16] Lopes N., Bergsland C.H., Bjørnslett M., Pellinen T., Svindland A., Nesbakken A., Almeida R., Lothe R.A., David L., Bruun J. (2020). Digital image analysis of multiplex fluorescence IHC in colorectal cancer recognizes the prognostic value of CDX2 and its negative correlation with SOX2. Lab. Investig..

[17] Pulsawatdi A.V., Craig S.G., Bingham V., McCombe K., Humphries M.P., Senevirathne S., Richman S.D., Quirke P., Campo L., Domingo E. (2020). A robust multiplex immunofluorescence and digital pathology workflow for the characterisation of the tumour immune microenvironment. Mol. Oncol..

[18] Elliott K., McQuaid S., Salto-Tellez M., Maxwell P. (2015). Immunohistochemistry should undergo robust validation equivalent to that of molecular diagnostics. J. Clin. Pathol..

[19] Mezheyeuski A., Bergsland C.H., Backman M., Djureinovic D., Sjöblom T., Bruun J., Micke P. (2018). Multispectral imaging for quantitative and compartment-specific immune infiltrates reveals distinct immune profiles that classify lung cancer patients. J. Pathol..

[20] Stack E.C., Wang C., Roman K.A., Hoyt C. (2014). Multiplexed immunohistochemistry, imaging, and quantitation: A review, with an assessment of Tyramide signal amplification, multispectral imaging and multiplex analysis. Methods.

[21] Koelzer V.H., Sirinukunwattana K., Rittscher J., Mertz K.D. (2019). Precision immunoprofiling by image analysis and artificial intelligence. Virchows Archiv.

[22] Lewis C., McQuaid S., Clark P., Murray P., McGuigan T., Greene C., Coulter B., Ki M., James J. (2018). The Northern Ireland Biobank: A Cancer Focused Repository of Science. Open J. Bioresour..

[23] Bankhead P., Loughrey M.B., Fernández J.A., Dombrowski Y., McArt D.G., Dunne P.D., McQuaid S., Gray R.T., Murray L.J., Coleman H.G. (2017). QuPath: Open source software for digital pathology image analysis. Sci. Rep..

[24] Humphries M.P., Hynes S.O., Bingham V., Cougot D., James J., Patel-Socha F., Parkes E., Blayney J.K., Rorke M.O., Irwin G. (2018). Automated Tumour Recognition and Digital Pathology Scoring Unravels New Role for PD-L1 in Predicting Good Outcome in ER-/HER2+ Breast Cancer. J. Oncol..

[25] Craig S.G., Humphries M.P., Alderdice M., Bingham V., Richman S.D., Loughrey M.B., Coleman H.G., Viratham-Pulsawatdi A., McCombe K., Murray G.I. (2020). Immune status is prognostic for poor survival in colorectal cancer patients and is associated with tumour hypoxia. Br. J. Cancer.

