# Supplementary material for: PD-L1 Multiplex and Quantitative Image Analysis for Molecular Diagnostics
Source: Cancers (Basel). 2020 Dec 23;13(1):29. doi: 10.3390/cancers13010029 (PMC7796246; doi:10.3390/cancers13010029)
Supplement: Supplementary file 1 [file cancers-13-00029-s001.pdf]

Supplementary Materials

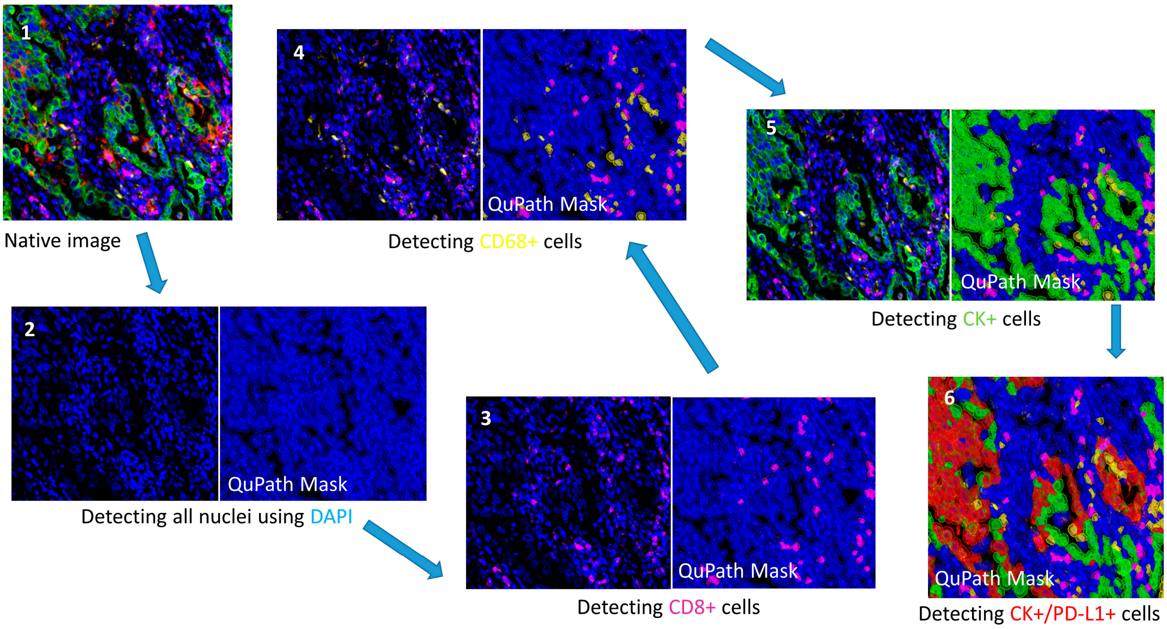

Figure S1. Flow diagram for mIF cell detection. Biomarker detection sequence with image analysis mask.

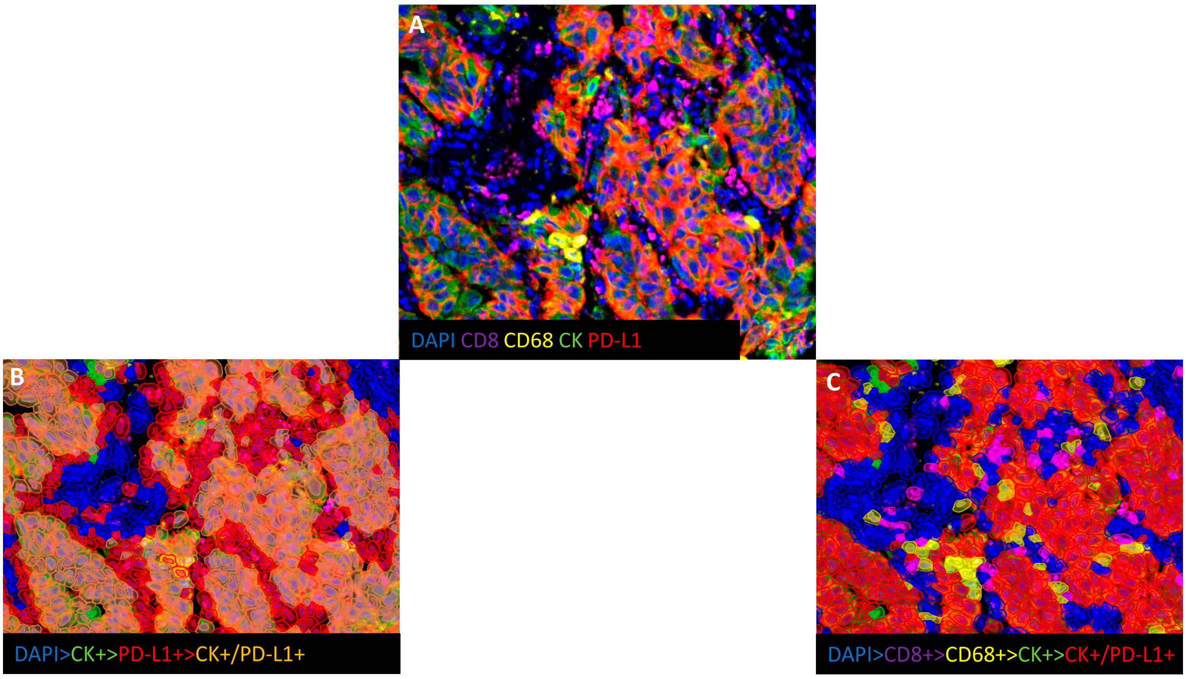

Figure S1. Importance of detection order. (**A**) The native multiplex image. (**B**) Image showing over detected PD-L1 signal in the CK due to incorrect phenotype classification sequence DAPI>CK+PD-L1+>CK+/PD-L1. (**C**) Accurate phenotypic classification sequence detecting CD8 and CD68 postive cells initially before assessing the PD-L1 channel. DAPI>CD8+>CK+>PD-L2+.

Table 1. Optimised retrieval methods and staining conditions.

Hybridisation Method	Biomarker	Clone	Source	Catalogue Number	Automated Platform	Epitope Retrieval	Dilution	Incubation	Detection Chemistry
DAB Single	PD-L1	SP263	Ventana	740-4907	Ventana	CC1 64 mins	Neat	20 mins , 37◦C	OptiView DAB Detection Kit
DAB Single	CD8	C8/144B	Dako	M7103	Bond Rx	ER2 20 minutes	1 in 400	30 minutes, ambient	Bond Polymer Refine + Enhancer
DAB Single	CD68	514H12	Novocastra	NCL-L-CD68	Bond Rx	ER2 20 minutes	1 in 200	30 minutes, ambient	Bond Polymer Refine + Enhancer
DAB Single	CK	AE1/AE3	Dako	M3515	Bond Rx	ER2 20 minutes	1 in 100	30 minutes ambient	Bond Polymer Refine + Enhancer
mIF position #1	PD-L1	SP263	Ventana	740-4907	Bond Rx	ER2 20 minutes	1:2	30 minutes, ambient	Opal 520 @1:150 for 30min
mIF position #2	CD8	C8/144B	Dako	M7103	Bond Rx	ER2 20 minutes	1 in 400	30 minutes, ambient	Opal 690 @1:150 for 30min
mIF position #3	CD68	514H12	Novocastra	NCL-L-CD68	Bond Rx	ER2 20 minutes	1 in 200	30 minutes, ambient	Opal 570 @1:150 for 30min
mIF position #4	CK	AE1/AE3	Dako	M3515	Bond Rx	ER2 20 minutes	1 in 100	30 minutes, ambient	Opal 620 @1:150 for 30min

Data S1. Bespoke Script for the Detection of Individual Biomarkers on Mif Images Using Qupath

```
measurement1 = "Cell: CD8 (Opal 690) mean" //CD8

measurement2 = "Nucleus: CD68 (Opal 570) mean" //CD68

measurement3 = "Nucleus: CK (Opal 620) mean" //CK

measurement4 = "Cell: PD-L1 (Opal 520) mean" //PD-L1



CD8 = getPathClass("CD8 (Opal 690)")

CD68 = getPathClass("CD68 (Opal 570)")

CK = getPathClass("CK (Opal 620)")

DAPI = getPathClass ("DAPI")

PDL1 = getPathClass("PD-L1 (Opal 520)")



//classify CD8

selectObjects {it.isDetection()}

for (detection in getSelectedObjects()) {

    m1 = measurement(detection, measurement1)



    if ( m1 > 2.5 )
```

```
        detection.setPathClass(CD8)

  else
  detection.setPathClass(DAPI)

}
fireHierarchyUpdate()

//classify CD68
selectObjects { p -> p.getPathClass() == getPathClass("DAPI")}
for (detection in getSelectedObjects()) {
    m2 = measurement(detection, measurement2)

    if ( m2 > 2 )
        detection.setPathClass(CD68)

  else
  detection.setPathClass(DAPI)
```

```
}
fireHierarchyUpdate()

//classify CK
selectObjects { p -> p.getPathClass() == getPathClass("DAPI")}
for (detection in getSelectedObjects()) {
    m3 = measurement(detection, measurement3)

    if ( m3 > 2.5 )
        detection.setPathClass(CK)

  else
  detection.setPathClass(DAPI)

}
fireHierarchyUpdate()
```

```
//classify PD-L1

selectObjects { p -> p.getPathClass() == getPathClass("CK (Opal 620)")}

for (detection in getSelectedObjects()) {

        m4 = measurement(detection, measurement4)



        if ( m4 > 1.5 )

                detection.setPathClass(PDL1)



    else

    detection.setPathClass(CK)



}

fireHierarchyUpdate()
```
